# Temperature induced phase transformation in Co

**DOI:** 10.1038/s41598-022-14302-x

**Published:** 2022-06-16

**Authors:** R. Sewak, C. C. Dey, D. Toprek

**Affiliations:** 1 Saha Institute of Nuclear Physics, 1/AF Bidhannagar, Kolkata, 700064 India; 2 Homi Bhabha National Institute, Anushaktinagar, Mumbai, 400094 India; 3 Vinca Institute of Nuclear Sciences, National Institute of the Republic of Serbia, University of Belgrade, P. O. Box 522, Belgrade, 11001 Serbia

**Keywords:** Condensed-matter physics, Structural materials, Theory and computation, Ferromagnetism, Magnetic properties and materials

## Abstract

Temperature dependent phase transformation behavior in cobalt from hexagonal close-packed (hcp) to face centered cubic (fcc) has been found to be contradictory to that reported earlier. It is found that hcp phase stabilizes at both low and high temperature ($\sim$873 K) while fcc phase is stabilized at $\sim$500 K. At 298 K, hcp Co has been found to be predominant ($\sim$70%) where hcp magnetic phase is $\sim$60%. At 973 K, hcp phase is again predominant ($\sim$73%), but it is mainly the non-magnetic phase ($\sim$67%). Contrary to present results, it was found earlier that fcc phase was stabilized at high temperature and hcp to fcc transformation occured at $\sim$700 K. Present results from perturbed angular correlation measurements, therefore, requires a new theoretical interpretation for Co phase transformation. From present measurements, hyperfine magnetic fields in Co at room temperature for the hcp and fcc phases have been found to be 18.7(6) and 12.8(3) T, much lower than earlier reported results. The hyperfine magnetic fields at $^{181}$Ta impurity atom have been calculated by density functional theory (DFT) employing the full potential (linearized) augmented plane wave method (FP-LAPW). Present calculated results for both hcp and fcc phases corroborate our experimental results.

## Introduction

The elemental cobalt (Co) is known to have ferromagnetism at room temperature. It has two crystal structures of hexagonal close-packed (hcp) and face centred cubic (fcc). At low temperature, Co crystallizes in a hcp lattice with a c/a ratio = 1.62 at 298 K, very close to the ideal value (c/a = 1.633) for closet packing. It was found^1^ that Co undergoes a phase transition from hcp to fcc just below 700 K. From hyperfine interaction studies of perturbed angular correlation (PAC) using $^{111}$In probe, Lindgren et al.^2^ reported that Co has a pure hcp phase at $\le 604$ K while it has a pure fcc phase at 771 K. In the intermediate 648 K, both hcp and fcc phases were found to co-exist^2^. In a subsequent report by Bedi et al.^3^ using the same hyperfine interaction method but with a different $^{181}$Hf probe, however, it was shown that the hcp phase was present up to 700 K and due to the sluggish nature of this phase transition, both hcp and fcc phases were found to be present at room temperature and above. From previous PAC measurements using $^{181}$Hf probe^3^, the hcp phase was found to be $\sim$66% at room temperature along with a significant fraction ($\sim$5–10%) for the fcc phase. The presence of fcc fraction at room temperature was confirmed also from X-ray diffraction pattern.

The magnetism of Co was found for both these phases^2,3^. At room temperature, the magnetic field strength for the hcp phase was found to be approximately 10% higher than the fcc phase^3^. It was reported that both the structures of hcp and fcc have room temperature ferromagnetism at the probe Ta impurity site^3^. From earlier reports^2,3^, a higher value of hyperfine magnetic field for the hcp phase was found compared to the fcc phase. From the previous report by Lindgren et al.^2^ no fcc phase fraction was found at room temperature while a definite value of hyperfine magnetic field for the fcc phase was reported by Bedi et al.^3^.

The temperature induced structural phase transition in Co from hcp to fcc is not properly understood. From previous reports^4–7^, it was found that magnetism plays an important role in the phase stability of 3d transition metals. From calculations by density functional theory (DFT)^8^, it was found that if Co was not magnetic, it would choose the fcc phase as its ground state. It was reported^7,9,10^ that absence of magnetism at high temperature induced this phase transition. Uhl and Kübler^7,11^, from theoretical calculations, showed that spin fluctuation and reduced magnetism at high temperatures lower the free energy of the fcc phase and triggers the hcp to fcc phase transition. From their calculations, they reported a transition temperature of 590 K by considering the magnetic effects only. But, it is recently shown^8^ that reduced magnetism alone is not sufficient to destabilize the hcp phase at high temperature. It was reported^8^ that vibrational energy arising due to vibration of ionic lattice is the main driving force for the hcp to fcc phase transition and besides this, other free energy terms like magnetic, electronic and volume effects must be included to understand this phase transition properly. By including all these contributions to free energy, they reported a transition temperature of 825 K. Therefore, to understand the main driving force that causes the hcp to fcc phase transition in Co, it is very important to determine the temperature induced phase transformation behavior. Considering discrepancies of results in the previous reports^2,3^, temperature dependent PAC measurements in Co using $^{181}$Hf probe have been re-investigated in the present report.

The Co is an unique element in the periodic table. It has a ferromagnetism at room temperature and possess both the hcp and fcc crystalline phases that transforms with temperature. From previous hyperfine interaction studies controversial reports on the existence of these phases with temperature were reported and its phase transformation behavior with temperature is not yet clearly understood. The temperature range in which the phase transformation occurs and the extent to which each fraction exists in temperature below and above the phase transition is still a matter of debate. From theoretical considerations, phase stabilities in Co were attributed to the presence or absence of magnetism. The importance of present studies lies in the fact that both the structural changes and the change in ferromagnetism with temperature can be studied simultaneously. This helps to understand the phase transformation behavior with temperature and the role of magnetism in the phase stability.

The hcp crystalline phase has a non-cubic symmetry and the charge distribution surrounding the probe generates an electric field gradient (EFG) at the probe impurity site. Therefore, a combined magnetic and electric quadrupole hyperfine interaction is expected in hcp Co at room temperature. Above Curie temperature, when there is no ferromagnetism, only a pure quadrupole interaction is expected. For the fcc phase, however, no hyperfine electric quadrupole interaction is expected above the Curie temperature and there will be no perturbation in the PAC spectrum.

The PAC is a useful nuclear technique for the studies of structural and magnetic phase transitions in materials^12–14^ where a suitable radioactive nucleus is inserted as a probe within the material. For present studies, we have used $^{181}$Hf probe decaying to $^{181}$Ta through $\beta ^-$. The daughter nucleus has a strong 133–482 keV $\gamma -\gamma$ cascade passing through the 482 keV intermediate level with spin angular momentum $I = {5/2}^+$, level half-life T$_{1/2}$ = 10.5 ns. This intermediate level also has high values of electromagnetic moments^15^ and produces strong extra-nuclear perturbations due to hyperfine interactions. In a non-cubic environment of the probe, the electric quadrupole moment of the probe interacts with the surrounding electric field gradient and in a magnetic material, the magnetic field interacts with the surrounding magnetic field. Due to these interactions, the selected angular correlation (133–482 keV cascade) is perturbed.

The perturbation function corresponding to static pure electric quadrupole interaction, for a polycrystalline sample and for $I=5/2$ intermediate level is given by^13,14^

1 $$\begin{aligned} G_{22}(t) = S_{20}(\eta )+\sum _{n=1}^3 S_{2n} (\eta ) cos (\omega _nt) exp(-\delta \omega _nt) \end{aligned}$$

The coefficients S$_{2n}$^16^ depend upon orientation of the electric field gradient (EFG) tensor and are different for single and polycrystalline samples. The parameter $\delta$ represents the Lorentzian shape damping of the quadrupole frequency distribution due to imperfections in crystal lattice structure (defects, impurities, etc.). The electric field gradient in the principle axis system can be described by two parameters. (i) The quadrupole frequency ($\omega _Q$) and asymmetry parameter $\eta$^16^.

If the probe nuclei experience a pure magnetic field (in a cubic crystalline environment), the corresponding magnetic perturbation can be described by

2 $$\begin{aligned} G_{22}(t) = 1/5[1 + 2cos(\omega _Lt) + 2cos(2\omega _Lt)] \end{aligned}$$

Here, $\omega _L$ is the Larmor precession frequency and is given by $\omega _L = g\mu _NB_{hf}/\hbar$. From the measured PAC spectrum ($G_{22}(t)$ vs *t*), values of $\omega _L$ can be determined. The g factor of the probe nuclear level is related to the nuclear magnetic moment by $\mu = gI$, $\mu$ is nuclear magnetic moment of the intermediate level. In the above relation, $\mu _N$ is the nuclear magneton and $B_{hf}$ is the effective hyperfine magnetic field experienced by probe nuclei in the sample.

In a non-cubic site environment, the probe nucleus experiences a combined magnetic dipole and electric quadrupole hyperfine interaction. The perturbation function due to this combined interaction depends on five interaction parameters and on the nuclear spin *I*^17–19^. In this case, the interaction parameters are the magnetic frequency $\omega _L$, the quadrupole frequency $\omega _Q$, the asymmetry parameter $\eta$, the Euler angles $\theta$ and $\phi$ which describe the relative orientation of the magnetic hyperfine field and the EFG tensor.

## Experimental details

For present PAC measurements, a four detector time differential perturbed angular correlation (TDPAC) set up with two LaBr$_3$(Ce) (38$\times$25.4 mm$^2$) detectors and two BaF$_2$ (50.8 × 50.8 mm$^2$) detectors have been used. Details about data collection and generation of A$_{22}$G$_{22}$(t) from the four coincidence spectra at 180$^{\circ }$ and 90$^{\circ }$ can be found in our earlier report^16^. The program WINFIT^20^ was used to fit the PAC spectra. In the fitting, we assume there is no interference on the magnetic hyperfine frequency by the electric quadrupole frequency. This is a special situation and can be justified by considering the fact that the Larmor precession frequency is much larger than the electric quadrupole frequency i.e. $\omega _L \gg \omega _Q$. In the analysis, we therefore consider the electric and magnetic frequency components are independent to each other and these different components are simply combined to get the total PAC spectrum.

The sample for PAC measurement was prepared by arc melting high purity (99.995%) cobalt wire with a tiny piece of active $^{181}$Hf wire in argon atmosphere. The probe concentration in the sample ($\sim$0.3 at.%) was too small to affect the bulk sample properties. The active $^{181}$Hf was produced from natural Hf by thermal neutron capture at Dhruba reactor (Mumbai), India.

## Results and discussion

**Table 1:** PAC results for Co at three selected temperatures.

Temp. (K)	Comp.	Quadrupole interaction				Magnetic dipole interaction		
		$\omega _Q$	$\eta$	$\delta$	*f*	$\omega _L$	$\delta$	*f*
		(Mrad/s)		($\%$)	($\%$)	(Mrad/s)	($\%$)	($\%$)
298	1	58.9 (4)	0.38 (2)	0	9 (1)			
	2	16 (1)	0	0	9 (1)			
	3					1154 (25)	27 (7)	65 (3)
	4					823 (7)	5 (2)	17 (1)
298$^\dagger$	1	59.5 (3)	0.39 (2)	0	10 (1)			
	2	13.8 (6)	0	0	9 (1)			
	3					1187 (24)	22 (5)	60 (3)
	4					810 (7)	9 (2)	21 (1)
873	1	67.5 (2)	0.16 (2)	2.5 (6)	64 (4)			
	2	17 (1)	0	0	11 (1)			
	3					935 (11)	0	5 (1)
	4					674 (3)	0	20 (3)
973	1	67.1 (2)	0.16 (2)	3.0 (6)	67 (4)			
	2	16 (1)	0	0	8 (1)			
	3					872 (8)	0	6 (1)
	4					676 (2)	0	19 (1)

$^\dagger$ after annealing the sample.

Components 1 and 3 assigned to hcp while 2 and 4 assigned to fcc phase.

**Figure 1:**
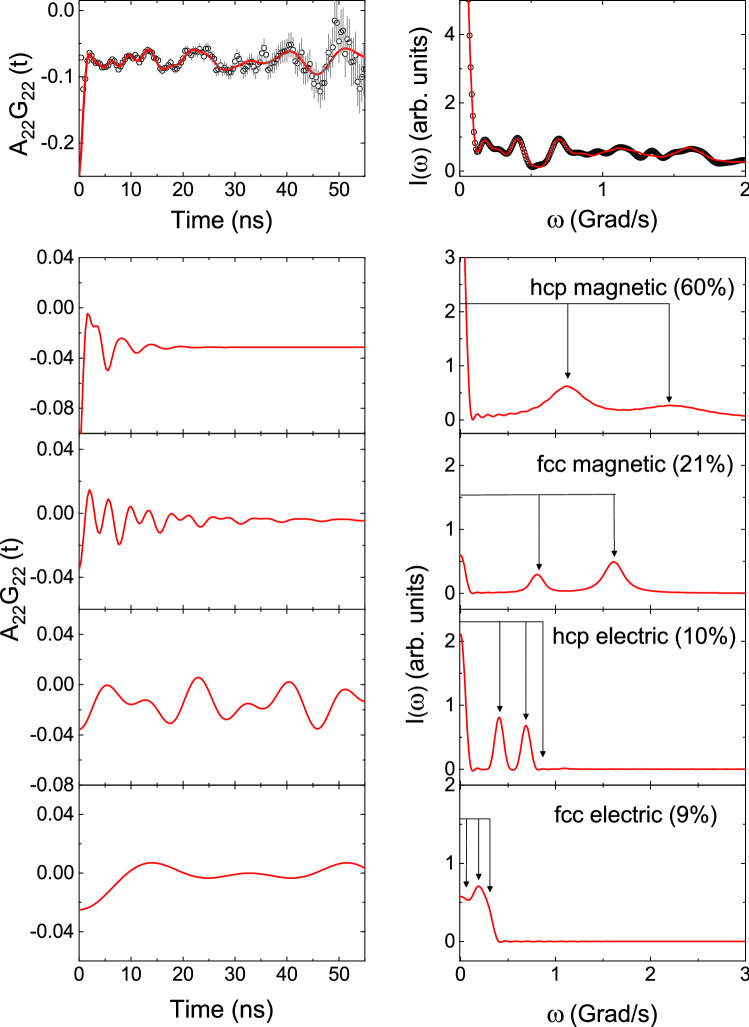
Total PAC spectrum at 298 K in annealed sample (top) and its decomposed spectra (bottom); Left panel shows time spectra and right panel shows corresponding Fourier transforms.

From PAC spectrum at room temperature four frequency components have been found. Results for the four components in as prepared sample and also in annealed sample are shown in Table 1. The sample was annealed at 773 K for one day. It is found that the PAC spectra and the results before and after annealing do not change appreciably. The PAC spectrum found after annealing the sample is shown in Fig. 1. In annealed sample, the major component ($\sim$60%) gives $\omega _L$ = 1187(24) Mrad/s, with a large value of frequency distribution width ($\delta \sim 22\%$ ). This has been attributed to hyperfine magnetic field corresponding to hcp Co. It is known that Co has a hcp crystal structure at room temperature and a ferromagnetism is expected to be stronger at low temperature. The magnetic frequency component with a lower value of $\omega _L$ has been attributed to fcc Co. It is already known^2,3^ that hyperfine magnetic field in fcc Co is lower than the hcp Co. The two quadrupole frequency components found have been assigned also to hcp and fcc crystal structures of Co. For annealed sample, the quadrupole frequency component with values of $\omega _Q = 59.5(3)$ Mrad/s, $\eta =0.39(2)$ , $\delta = 0$ has been attributed to hcp Co and the lower frequency component (Table 1) with values of $\omega _Q = 13.8(6)$ Mrad/s, $\eta = 0$, $\delta = 0$ has been attributed to fcc Co. A pure fcc Co should not produce any EFG. But, in this case, we are measuring the EFG at the probe impurity site where the local site symmetry is broken in presence of impurity and a non-cubic symmetry is generating a finite EFG. The decomposed four spectra corresponding to these four components are shown in Fig. 1. The hcp magnetic component is clearly found to be the predominant component. But, the hcp electric quadrupole component and the two fcc components (magnetic and electric quadrupole) are clearly observed at room temperature. The total fcc phase at room temperature has been found to be quite large ($\sim$28%). The results in pre-annealed sample are also found to be similar (Table 1).

**Figure 2:**
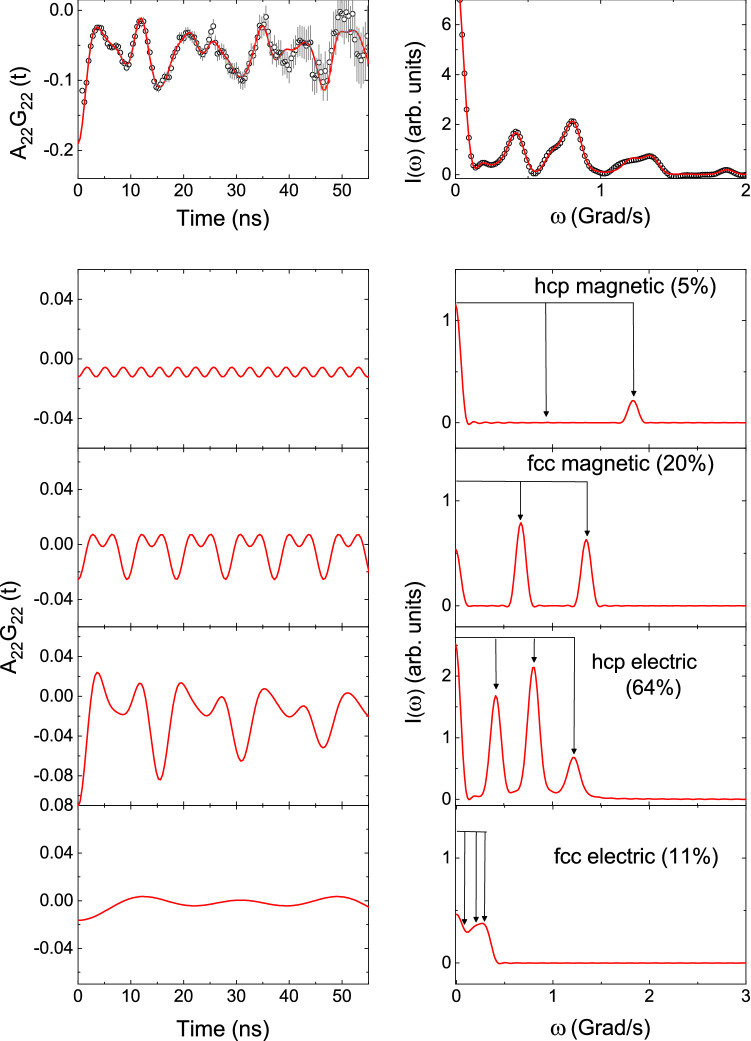
Total PAC spectrum at 873 K (top) and its decomposed spectra (bottom); Left panel shows time spectra and right panel shows corresponding Fourier transforms.

At room temperature, the fcc fraction in Co was reported to be present by Bedi et al.^3^ while it was not found by Lindgren et al.^2^. From X-ray diffraction studies, it was reported to be present with a small fraction (5–20%) at room temperature. The present results of phase fractions contradict with the results reported in reference^2^.

The magnitude of hyperfine magnetic field strengths for the hcp and fcc phases at room temperature have been found to be 18.7(6) T and 12.8(3) T (absolute values), respectively for the present measured values of $\omega _L$ in annealed sample (considering a value of g = 1.31(3)^15^ for the 482 keV level). While the magnetic field strength for the hcp phase is higher than the fcc phase as reported earlier^2,3^, the present measured value of hyperfine magnetic field for the hcp phase is only $\sim$46% to that reported by Bedi et al.^3^ and for the fcc phase, it is only $\sim$35%. The corresponding hyperfine magnetic fields found from previous measurement^3^ were 40.7 and 36.3 T for hcp and fcc phases, respectively. It can be mentioned here that the internal hyperfine magnetic fields determined by the PAC technique represent the absolute values only. The sign of the fields can not be determined unless an external magnetic magnetic field is applied.

**Figure 3:**
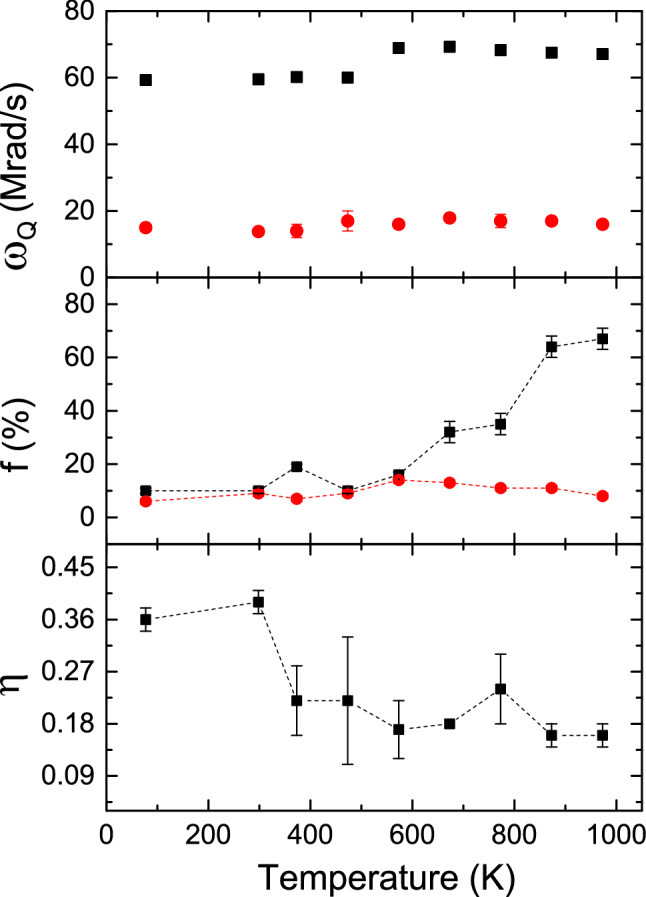
Variations of $\omega _Q$, site fraction (f) and $\eta$ with temperature for the hcp (filled square) and fcc (filled circle) phases.

**Figure 4:**
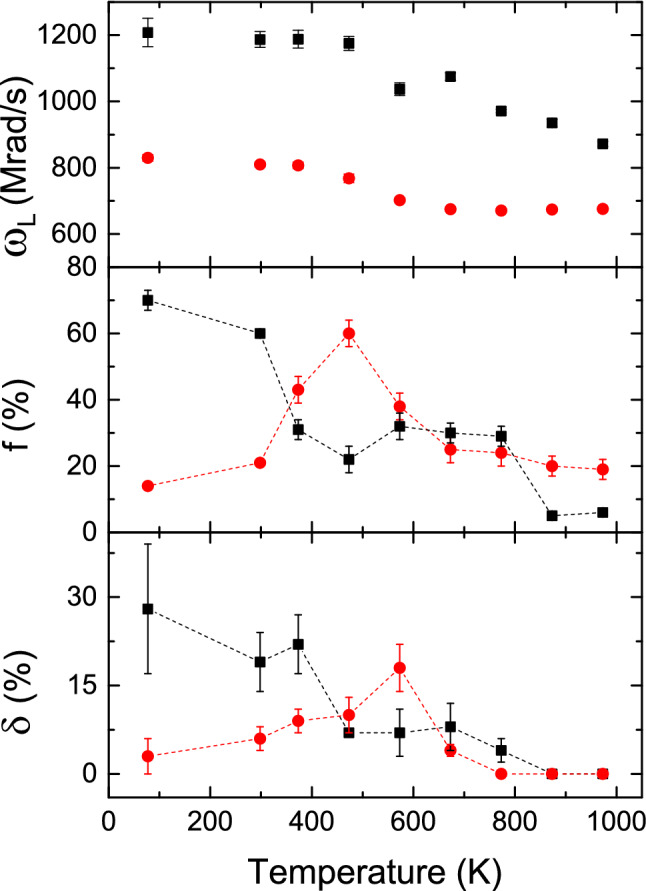
Variations of $\omega _L$ along with their site fraction (f) and $\delta$ with temperature for the hcp (filled square) and fcc (filled circle) phases.

To determine the variations of hyperfine magnetic fields and electric field gradients with temperature, we have done measurements at a lower temperature of 77 K and in the higher temperature range (373–973 K) have been carried out. At 77 K also, these four components have been observed and the results do not change much compared to the results found at room temperature. Interestingly, at high temperature ($\ge 873$ K), a drastic change in the PAC spectrum has been found (Figure 2). At 873 K, all four components have been found but, their site fractions are completely changed (Table 1). At this temperature, the hcp quadrupole frequency component is predominant ($\sim$64%) while the hcp magnetic component fraction has been found as a negligible fraction ($\sim$5%) as shown in Fig. 3. The results remain almost same at 973 K. At temperatures $\sim$500 K, on the other hand, the hcp magnetic fraction is much lower than the fcc magnetic fraction (Figure 4).

**Figure 5:**
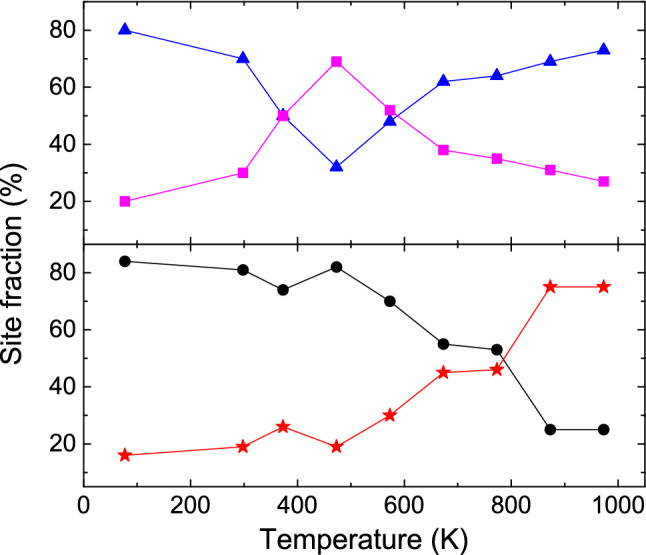
Top: Variations of site fractions for total hcp (triangle) and total fcc (square) phases with temperature. Bottom: Variations of site fractions for total magnetic (circle) and total non-magnetic (star) components with temperature.

In the temperature range 77-973 K, variations of $\omega _Q$, $\eta$ and site fractions with temperature for the hcp and fcc phases of Co are shown in Fig. 3. For both these fractions, $\delta$ were found to be zero up to 773 K. At 873 and 973 K, strength of quadrupole interaction increases with small values of frequency distribution width. Variations of $\omega _L$, $\delta$ and corresponding site fractions with temperature for the two magnetic phases are shown in Fig. 4. It is found that at room temperature, the hcp magnetic fraction is predominant but at 973 K, the hcp quadrupole fraction is predominant. Values of $\omega _Q$ for both hcp and fcc components do not change in the temperature range 77–500 K. However, for the hcp component, it shows a clear discontinuity at $\sim$500 K and at this temperature the value of $\omega _Q$ increases by approximately 10% compared to the room temperature value. In the temperature range 500-973 K, the enhanced values of $\omega _Q$ remain unchanged. This change in quadrupole frequency can be understood by the fact that from around 500 K, the total electric quadrupole fraction increases rapidly at the expense of magnetic fraction (Fig. 5). Probably, at lower temperatures (T $\le 500$ K), the quadrupole frequencies have been modified by the stronger magnetic components. At temperatures T > 500 K, the magnetic component fraction is not sufficiently strong to modify the quadrupole frequency of the hcp component. For the fcc component, this is not clearly observed because of lower values of $\omega _Q$, although it shows an increasing tendency in the higher temperature region (500–973 K).

Values of $\omega _L$ are found to clearly decrease with temperature as expected. For the hcp phase, $\omega _L$ decreases with temperature in the high temperature region (above 400 K). For the fcc phase, however, $\omega _L$ remains almost same in the temperature range 600–973 K. This is quite unexpected and the reason is not clearly understood. A vanishingly small site fraction for the hcp magnetic field at 973 K indicates that the Curie temperature for this phase is $\sim$975 K and it agrees well with the value of T$_c$ reported earlier^3^. For the fcc phase, however, a relatively large site fraction for the fcc magnetic component at 973 ($\sim$19%) has been found suggesting a higher Curie temperature for the fcc phase as found earlier.

Variations of total site fractions (magnetic and non-magnetic component together) with temperature for the hcp and fcc phases are shown in Fig. 5. It is found that the total hcp fraction first decreases with temperature and shows a minimum at $\sim$500 K. Above 500 K, the hcp fraction increases again with temperature and found to be maximum at around 973 K. On the other hand, the total fcc site fraction is $\sim$30% at room temperature. Up to 500 K, this phase increases with temperature and then decreases again. The total fcc fraction has been found to be maximum ($\sim$70%) at 473 K. At $\sim$873 K and above, the fcc phase again shows as a minor fraction. The Figure shows that at low temperature and also at high temperature (above 873 K), the hcp is found as a predominant phase while in the temperature range 373–573 K, the fcc phase is found to be more stable than the hcp phase. After measurement at 973 K, a remeasurement at 298 K was carried out where the results are found to be reversible. Variations of total magnetic (hcp and fcc together) and non-magnetic fractions with temperature are also shown in Fig. 5. It is found that up to 500 K, the total magnetism does not change much. After $\sim$500 K, the magnetic component decreases and the non-magnetic component increases with temperature. So, in the temperature region (77-500K) where the total magnetism remains almost unchanged, the phase fraction changes from hcp to fcc. Also, when the magnetism is low at high temperature ($\ge 873$ K), the hcp phase fraction again shows its maximum value. From present results it appears that magnetism has no role in the phase stabilization.

Present results contradict with the results reported earlier^2^, where a pure fcc magnetic phase was found at temperatures $\ge 771$ K and also contradict with the behavior of phase fractions with temperature as reported by Bedi et al.^3^. The reason for discrepancies could be attributed to experimental inaccuracy and also to wrong analysis of data in previous reports. In fact, determination of correct results from the much complicated PAC spectra where there are four frequency components (two magnetic and two quadrupole) and finding their variations with temperature are really challenging. Unless the data analysis are correctly done, results can be found which are far from reality. In present case, much efforts have been given to determine the results correctly. It can be mentioned here that from PAC measurements only, simultaneous variations of magnetic and non-magnetic components with temperature for the two phases can be determined and, therefore results from PAC measurements, can be considered as more accurate than other experimental techniques. The results reported by Bedi et al.^3^ are difficult to understand from following considerations. Starting a pure Co and using $^{181}$Hf probe, these authors^3^ reported a pure quadrupole interaction due to production of intermetallic compound Hf$_2$Co$_7$ at 700 K and a different compound of Hf$_6$Co$_{23}$ at 900 K. But, productions of these intermetallic compounds at high temperature seems to be improbable. This is because, (i) in their samples, the Hf concentrations were $\sim$0.2 at% and at this very small Hf concentration, production of any intermetallic compound with Co is unlikely. (ii) If any intermetallic compound is locally formed with Co, it should not be the pure single component, rather it should be a minor component, major component should be due to Co. (iii) If it is assumed that the Hf$_2$Co$_7$ is formed at 700 K, it is not understood why it transforms to another compound Hf$_6$Co$_{23}$ at 900 K and then again to elemental Co at 1300 K (not consistent with the reported phase diagram also). (iv) From our recent PAC measurements in Hf$_2$Co$_7$ and Hf$_6$Co$_{23}$^21,22^, completely different PAC spectra and results have been found than reported by Bedi et al.^3^. Due to these reasons, probably, the earlier measured hyperfine magnetic fields at Ta impurity (40.7 T and 36.3 T for hcp and fcc phases, respectively) are not reliable. Here, we are comparing the absolute values of hyperfine magnetic fields only as present measurements allow to determine the magnitudes only not the signs of magnetic fields.

## DFT calculation

To confirm our experimental results, we have calculated the hyperfine electric field gradients and magnetic fields at the $^{181}$Ta impurity site by density functional theory (DFT) employing the WIEN2K code in the framework of full potential (linearized) augmented plane wave method. The details about the calculation is given in the following section. Our calculated and experimental results are shown in Table 2. The calculated results for the site Hf-Ta1 are shown in the Table for comparing with our experimental results. It is found that for the hcp phase, the calculated result of $V_{zz}$ perfectly agree with our experimental value at room temperature although the calculated result of asymmetry parameter ($\eta \sim 0.7$) is higher that the experimentally observed value ($\eta \sim 0.4$). Here, the absolute values of $V_{zz}$ and $B_{hf}$ have been compared as present PAC experiments allow to determine the magnitude of the fields only. For the fcc phase also, the calculated result of $V_{zz}$ is in good agreement with our experimental value. In this case, a zero value of asymmetry parameter is found experimentally as well as from DFT calculation. For the hcp hyperfine magnetic field, the calculated result comes out to be $-25.75$ T (at 0 K) which is comparable to the value found experimentally at room temperature 18.7 T. However, if we extrapolate our experimental results of $\omega _L$ for hcp and fcc phases to 0 K with the power law formula given by

3 $$\begin{aligned} \omega _L(T) = \omega _L(0){\left[ 1-\frac{T}{T_c}\right] }^\beta \end{aligned}$$

with $\beta =0.42$, a value of $B_{hf}(0) = 20.9$ T (for $\omega _L= 1310$ Mrad/s) is obtained for hcp phase which is more closer to the calculated result. For the fcc phase also, the present calculated result at 0 K ( $-19.99$ T) is closer to the experimental result extrapolated to 0 K (13.3 T for $\omega _L= 837$ Mrad/s). On the other hand, a value of hyperfine magnetic field for the hcp phase at 9 K ($B_{hf}= -40.7$ T) was reported by Bedi et al.^3^ which is far away from the present calculated result. For the fcc phase also, the hyperfine magnetic field reported by Bedi et al.^3^ is much higher ($-36.3$ T at 9 K) than the calculated result. The present DFT calculated results of EFG and magnetic fields for both hcp and fcc phases, on the other hand, confirm our experimental results.

**Table 2:** Comparison of calculated and experimental results of hyperfine $V_{zz}$ and $B_{hf}$ for hcp and fcc phases in Co at $^{181}$Ta impurity site.

Phase	$V_{zz}$ ($10^{21}$ V/m$^2$)		$\eta$		$B_{hf}$ (T)	
	Cal.$^\dagger$	Exp.$^\ddagger$	Cal.$^\dagger$	Exp.$^\ddagger$	Cal.$^\dagger$	Exp.$^\S$
hcp	$-$ 6.64	6.67	0.71	0.39	$-$ 25.75	20.9
fcc	0.9543	1.55	0.00	0	$-$ 19.99	13.3

$$^\dagger $$At 0 K.

$^\ddagger$For annealed sample at 298 K.

$^{\S}$For annealed sample extrapolated to 0 K.

In this paper, we have performed the first-principles density functional theory (DFT) to calculate electric field gradients in term of largest diagonal component $V_{zz}$ and the asymmetry parameters $\eta$ in the pure Co as well as at Ta probe positions in the hcp and fcc crystal structure of Co. The hyperfine magnetic fields ($B_{hf}$) have also been calculated at the Ta probe positions and for no probe condition. The lattice parameters $a, b, c, \alpha , \beta$ and $\gamma$, and the fractional coordinates of crystallographic non-equivalent positions of hcp *P*63/*mmc* (space group number: 194) and fcc $Fm{\overline{3}}m$ (space group number: 225) crystal structure of Co are presented in the Table 3.

**Table 3:** The lattice parameters a, b and c (given in Å) and the fractional coordinates of crystallographic non-equivalent positions of hcp *P*63/*mmc* (space group number: 194) and fcc $Fm{\overline{3}}m$ (space group number: 225) crystal structure of Co getting by WIEN2k code^23^ (second and third columns) and these parameters taken from other research papers (fourth and fifth columns).

Crystal	WIEN2K calculation		Previous results^24,25^	
par.	hcp	fcc	hcp	fcc
a (Å)	2.4850	3.5283	2.5071	3.5442
b (Å)	2.4850	3.5283	2.5071	3.5442
c (Å)	4.0327	3.5283	4.0686	3.5442
$\alpha , \beta , \gamma$	90, 90, 120	90, 90, 90	90, 90, 120	90, 90, 90
x	0.3427	0.0030	0.3333	0.0000
y	0.6588	0.0020	0.6667	0.0000
z	0.2500	0.0000	0.2500	0.0000

At first, we have optimized the structural parameters. We have employed WIEN2k^23^ code where the full potential (linearized) augmented plane waves method (FP-LAPW) is implemented. The muffin-tin radii for Co was 1.6 a.u. The spherical harmonics inside the spheres are expanded up to $l_{max}=10$ while the charge density is Fourier expanded up to $G_{max}=16$. The energy convergence has been achieved by expanding the basis function up to $R_{MT}.K_{max}=7$, where $R_{MT}$ is the smallest atomic sphere radius in the unit cell and $K_{max}$ gives the magnitude of the largest $\vec {k}$ vector in the plane wave expansion. The generalized gradient approximation (GGA) parametrized by Perdew-Burke-Ernzerhof (PBE)^26–28^ was used for treated the electronic exchange-correlation energy. The energy of $-7$ Ry was set to separate core and valence states. It was selected a 8$\times$8$\times$8 k point mesh (for fcc structure) and 8$\times$8$\times$6 k point mesh (for hcp structure) to sample the entire Brillouin-zone (BZ), yielding 29 points (for fcc structure) and 40 points (for hcp structure) in the irreducible Brillouin-zone. The structure was relaxed according to Hellmann-Feynman forces calculated at the end of each self-consistent cycle, until the forces acting on all atoms were less than 0.068 eV/Å(5 mRy/a.u.)^29^. The self-consistency was achieved by demanding the convergence of the integrated charge difference between last two iterations to be smaller than $10^{-5} e$. All calculations were spin-polarized and refer to zero temperature.

**Figure 6:**
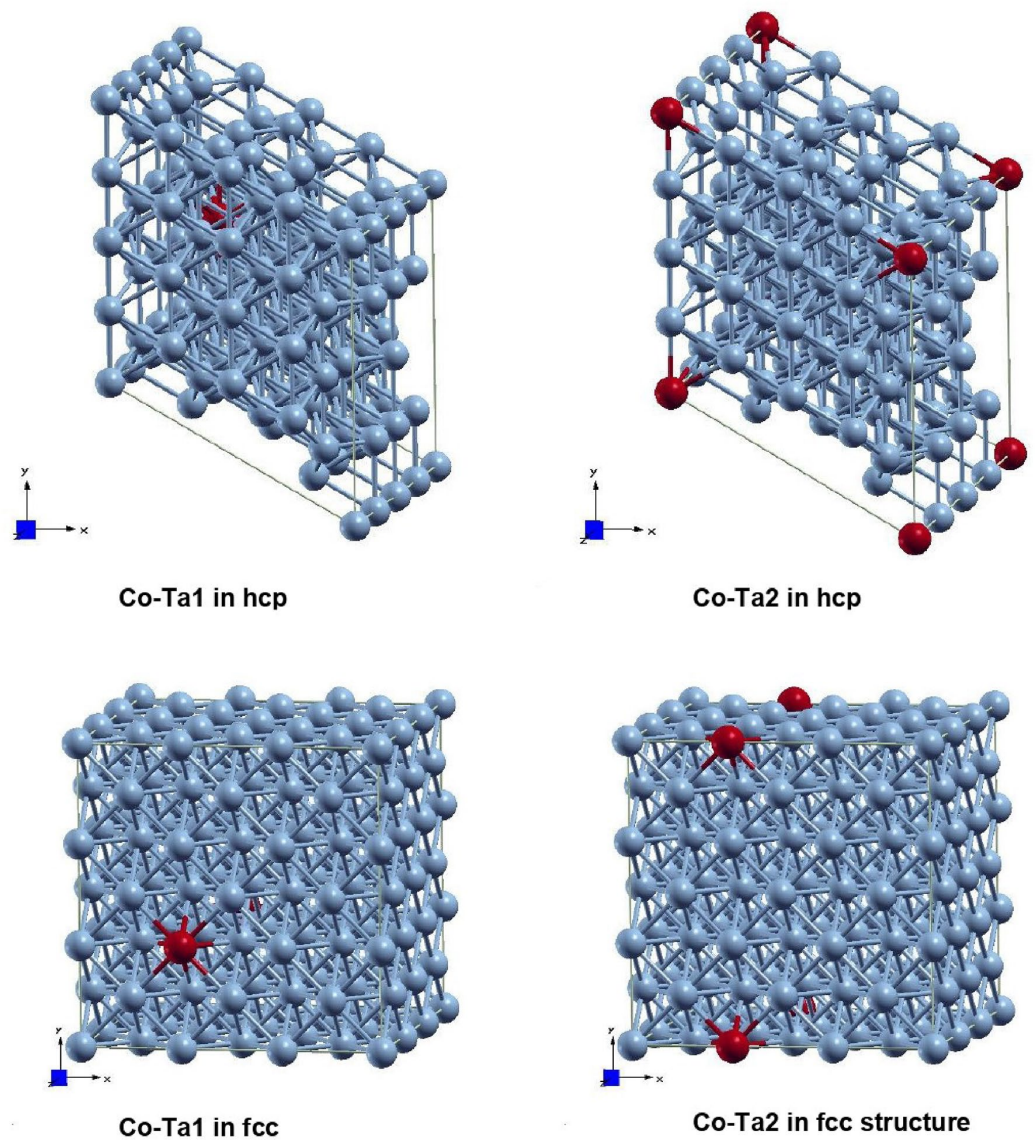
The different supercell models with a Co atom (blue colour) substituted by Ta probe (maroon colour) used for DFT calculations.

The theoretically optimized lattice parameters $a, b, c, \alpha , \beta$ and $\gamma$ and fractional coordinates of atoms are presented in Table 3 (second and third column). From Table 3, it can be seen that our calculated parameters are in very good agreement with the previous results (fourth and fifth column). After obtaining the optimized structural parameters, we construct 3$\times$3$\times$3 supercell (for fcc structure) and 4$\times$4$\times$4 supercell (for hcp structure) as shown in Fig. 6. To simulate a dopant in the crystal lattice we replaced Co by a Ta atom at the non-equivalent host site of Co, preserving the point group symmetry around the original atom. Consequently, the number of non-equivalent positions increased. The number of non-equivalent positions for fcc structures with 3$\times$3$\times$3 supercell is now 10 and for hcp structure with 4$\times$4$\times$4 supercell is now 25. Due to the local group symmetry in our calculations, we do not need to consider all of these non-equivalent positions. In fact, it is enough to consider 2 non-equivalent positions for fcc and for hcp structures. These substitutional structural positions have been marked as Co-Ta1 and Co-Ta2 (Fig. 6). We have checked that the two Ta atoms are sufficiently far from each other ($\sim$10 Å) to avoid significant impurity-impurity interactions. We have repeated the calculations for each substitutional structure, keeping all parameters and charge convergence criteria same as in the case of pure compounds, except that we have now selected a 3$\times$3$\times$3 k point mesh (for fcc structure) and 5$\times$5$\times$3 k point mesh (for hcp structure) to sample the entire Brillouin-zone (BZ), yielding 14 points (for fcc structure) and 15 points (for hcp structure) in the irreducible Brillouin-zone.

The calculated values of $V_{zz}$, asymmetry parameter ($\eta$) and the strength of the hyperfine magnetic field ($B_{hf}$) in the pure compounds as well as at Ta probe positions in the fcc and hcp Co structures are given in Table 4. The calculation of EFG were performed by using the method developed in Ref.^30^; which is implemented in WIEN2k code.

**Table 4:** The calculated $V_{zz}$ values in units of $10^{21}$ V/m$^2$, the asymmetry parameter $\eta$ values and the strength of the hyperfine magnetic field $B_{hf}$ for cobalt hcp *P*63/*mmc* and fcc $Fm{\overline{3}}m$ crystal structure.

Probe	Lattice Site	$V_{zz}$	$\eta$	$B_{hf}$(T)
**hcp** *P*63/*mmc*				
No probe (pure comp.)	Co1	− 0.18	0.00	− 8.73
$^{181}$Ta	Co-Ta1	− 6.64	0.71	− 25.75
	Co-Ta2	− 2.25	0.00	− 31.97
**fcc** $Fm{\overline{3}}m$				
No probe (pure comp.)	Co1	–	–	− 8.00
$^{181}$Ta	Co-Ta1	0.9543	0.00	− 19.99
	Co-Ta2	0.024	0.17	− 21.85

## Conclusion

The present report describes the hcp-fcc phase transformation behavior with temperature in the range 77–973 K by the PAC technique using $^{181}$Hf probe. Both hcp and fcc crystal structures of Co have been found to be present in the temperature range 77–973 K. At room temperature, the hcp magnetic fraction is found to be dominant. The total hcp fraction is also found to be maximum at low temperature (298 K and below) as expected. But, at high temperature ($\ge 873$ K), the hcp phase is again found to be predominant which is unexpected from existing knowledge and also from theoretical considerations. The fcc phase is found to be stabilized in the intermediate temperature ($\sim$500 K) where the ferromagnetism does not change much compared to the room temperature value. It is found that at high temperature, the nonmagnetic hcp phase is more stable than the ferromagnetic fcc phase and contradicts with the results found from theoretical calculations^8^. It appears that magnetism does not play any role in the phase stabilization of Co and some other mechanism is responsible for its temperature dependent phase transformation behavior. Therefore, to explain the hcp phase stability at high temperature, and the transformation of phases in the temperature range (77–500 K) where there is almost no changes of magnetism, present experimental results demand a new theoretical interpretation which remains unexplored till now. As a future outlook, it will be interesting to extend the PAC measurements at more higher temperature range ($> 973$ K) to observe whether the hcp phase remains dominant at this high temperature or to determine at which temperature the fcc phase takes over the hcp phase.

## Data Availability

The datasets generated during and/or analysed during the current study are available from the corresponding author on reasonable request.
